# Vitamin D and Graves’ Disease: A Meta-Analysis Update

**DOI:** 10.3390/nu7053813

**Published:** 2015-05-21

**Authors:** Mei-Yan Xu, Bing Cao, Jian Yin, Dong-Fang Wang, Kai-Li Chen, Qing-Bin Lu

**Affiliations:** 1 Department of Nutrition, Aerospace Center Hospital, Beijing 100049, China; E-Mail: 13011068860@163.com; 2 School of Public Health, Peking University, Beijing 100191, China; E-Mails: caobing@bjmu.edu.cn (B.C.); wdf@bjmu.edu.cn (D.-F.W.); 3 Department of Respiratory, Aerospace Center Hospital, Beijing 100049, China; E-Mail: chen_kaili@sina.cn

**Keywords:** Vitamin D, Graves’ disease, meta-analysis, sensitivity analysis, meta-regression

## Abstract

The association between vitamin D levels and Graves’ disease is not well studied. This update review aims to further analyze the relationship in order to provide an actual view of estimating the risk. We searched for the publications on vitamin D and Graves’ disease in English or Chinese on PubMed, EMBASE, Chinese National Knowledge Infrastructure, China Biology Medical and Wanfang databases. The standardized mean difference (SMD) and 95% confidence interval (CI) were calculated for the vitamin D levels. Pooled odds ratio (OR) and 95% CI were calculated for vitamin D deficiency. We also performed sensitivity analysis and meta-regression. Combining effect sizes from 26 studies for Graves’ disease as an outcome found a pooled effect of SMD = −0.77 (95% CI: −1.12, −0.42; *p* < 0.001) favoring the low vitamin D level by the random effect analysis. The meta-regression found assay method had the definite influence on heterogeneity (*p* = 0.048). The patients with Graves’ disease were more likely to be deficient in vitamin D compared to the controls (OR = 2.24, 95% CI: 1.31, 3.81) with a high heterogeneity (*I*^2^ = 84.1%, *p* < 0.001). We further confirmed that low vitamin D status may increase the risk of Graves’ disease.

## 1. Introduction

Graves’ disease is an autoimmune thyroid disease characterized in its typical presentation by the unique association of thyrotoxicosis, goiter and ophthalmopathy [1]. As one of the most frequent diseases among autoimmune disorders, an annual incidence of Graves’ disease was approximately 14 per 100,000 [2].

Graves’ disease is a multifactorial disease caused by a complex interaction between genetic and environmental factors that lead to the loss of immune tolerance to thyroid antigens, and therefore to the initiation of an immune reaction against the thyroid. For example, vitamin D receptor (VDR) gene polymorphisms were found to be associated with the risk for Graves’ disease [3].

Several studies also explored the association between vitamin D levels and Graves’ disease [4,5,6,7,8,9,10,11,12,13,14,15,16,17]. The results of some studies demonstrated that the patients with Graves’ disease had lower vitamin D levels or higher prevalence of vitamin D deficiency, suggesting that low levels of serum vitamin D was associated with Graves’ disease [5,6,7,8,10,11,12,13,14,15], while other studies did not find that vitamin D deficiency increased the risk of Graves’ disease [4,9]. The association between vitamin D levels and Graves’ disease is still under debate. Wang *et al.* [18] conducted the meta-analysis of the association between vitamin D and autoimmune thyroid disease, including the Graves’ disease with 13 studies [13,19,20,21,22,23,24,25,26,27,28,29,30,31]. However, the study [18] neither included enough references nor analyzed the sources of high heterogeneity. So the relationship needs to be further evaluated.

This update review aims to analyze the association between vitamin D levels and Graves’ disease in order to provide an actual view of estimating the risk.

## 2. Methods

### 2.1. Search Strategy

We searched for publications on vitamin D and Graves’ disease in English or Chinese. PubMed, EMBASE, Chinese National Knowledge Infrastructure (CNKI), China Biology Medical (CBM) and Wanfang databases were searched (up to April 15 2015) by two investigators, independently. The MeSH search terms were adapted: “Vitamin D”, “25 Hydroxyvitamin D”, “25 Hydroxyvitamin D_3_” in combination with “autoimmune thyroid disease” or “Graves’ disease”.

### 2.2. Inclusion/Exclusion Criteria

All the studies identified were reviewed independently by two investigators and the studies were included if they fulfilled the following criteria: (1) a case-control study or cohort study was reported; (2) the cases were diagnosed as Graves’ disease and the control group was composed of healthy individuals; (3) quantitative or qualitative vitamin D levels were described in the references. The references were excluded from the meta-analysis as follows: (1) the study was not related to Graves’ diseases; (2) the reference did not describe the vitamin D level in the two groups; (3) the reference consisted of duplicate data or publications from the included studies. The Newcastle-Ottawa Quality Assessment Scale was used to assess the quality of the studies included in the meta-analysis and performed by two reviewers with a third reviewer consulted in case of discrepancy.

### 2.3. Data Extraction

The information were extracted from the included studies using a standard form by the two reviewers. Any disagreement was resolved by discussion between the two reviewers. If consensus could not be reached, a third reviewer was consulted. The standard form included the variables as follows: the author, publication years, the study period, the study country, the mean age of case and control groups, the sample sizes of two groups, assay method of vitamin D, the detection index, the vitamin D concentrations (ng/mL) in the two groups (nmol/L to ng/mL by dividing by 2.5) or the number of vitamin D deficiency in the two groups and the cutoff for defining vitamin D deficiency.

### 2.4. Outcomes Measures

The primary outcome was the vitamin D level; the secondary outcome was the vitamin D deficiency. Comparisons were performed between the Graves’ cases and control population.

### 2.5. Statistical Analysis

The median and range were used to estimate the mean and variance with the method by Hozo *et al.* [32]. Heterogeneity among the studies was assessed using the Cochran Q and the *I*^2^ statistic. For the Q statistic, *p* < 0.10 indicates statistically significant heterogeneity. For the *I*^2^ statistic, *I*^2^ > 50% indicates a large heterogeneity. Fixed-effects model with Mantel-Haenszel method was used if Q statistic (*p* < 0.10) or *I*^2^ < 0.05; otherwise, random effects model was used. The standardized mean difference (SMD) and 95% confidence interval (CI) were calculated for the primary outcome. Pooled odds ratio (OR) and 95% CI were calculated for the secondary outcome. In case of heterogeneity, subgroup analysis was conducted. The Egger weighted regression test was used to statistically assess publication bias (*p* < 0.05 was considered as indicative of statistically significant publication bias). All statistical analyses were performed using Stata 12.0 (Stata Corp LP, College Station, TX, USA).

## 3. Results

**Figure 1:**
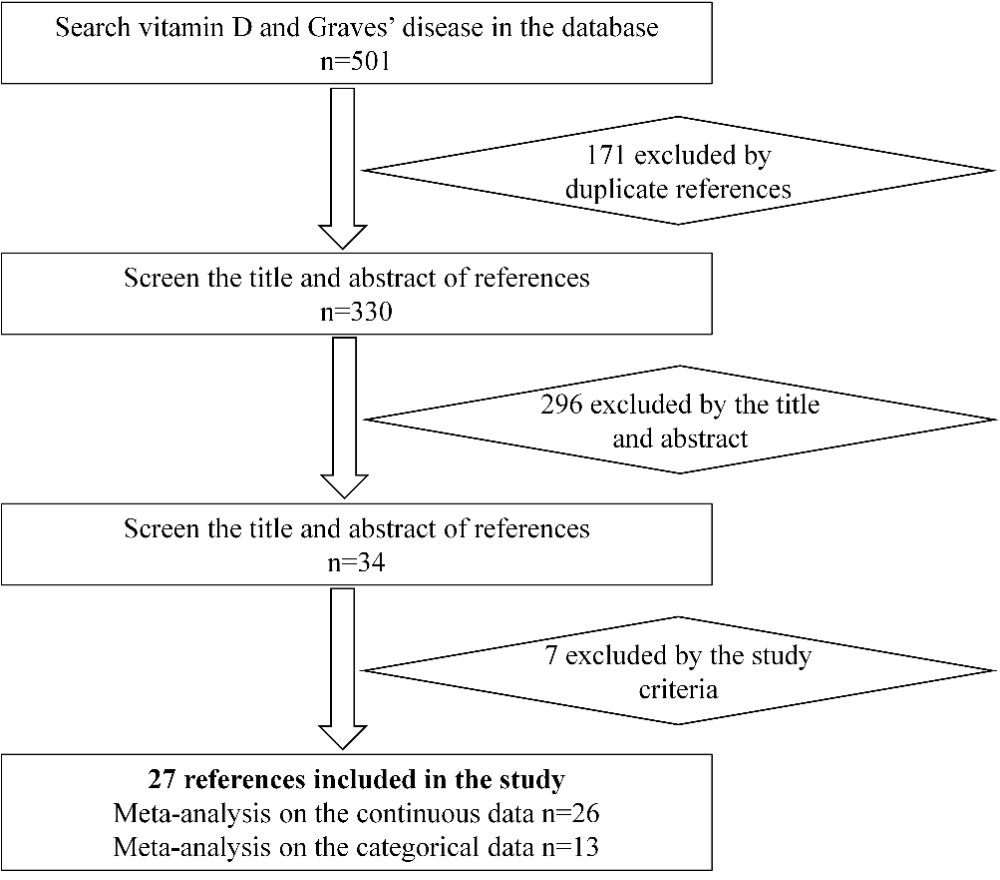
The flow diagram of the study selection.

In our study, we initially searched 501 related references, among which 171 were duplicates. When removing the duplicates and other unrelated references (insufficient data about the vitamin D and disease definition unrelated to the Graves’ disease in the references), 27 references met our inclusion criteria and were recruited in the meta-analysis (Figure 1). According to the data type, 26 and 13 references were used as continuous data and categorical data on the meta-analysis of vitamin D and Graves’ disease, respectively.

### 3.1. Information of the Included Studies

The information of the included studies were listed in Table 1. Most studies were published after 2012 (63.0%, 17/27) and from China (66.7%, 18/27). There were 3716 study subjects, including 1770 (47.6%) cases with Graves’ disease and 1946 (52.4%) controls. All studies received a score of more than or equal to six, indicating good quality.

### 3.2. Continuous Data of Vitamin D Level and Graves’ Disease

**Figure 2:**
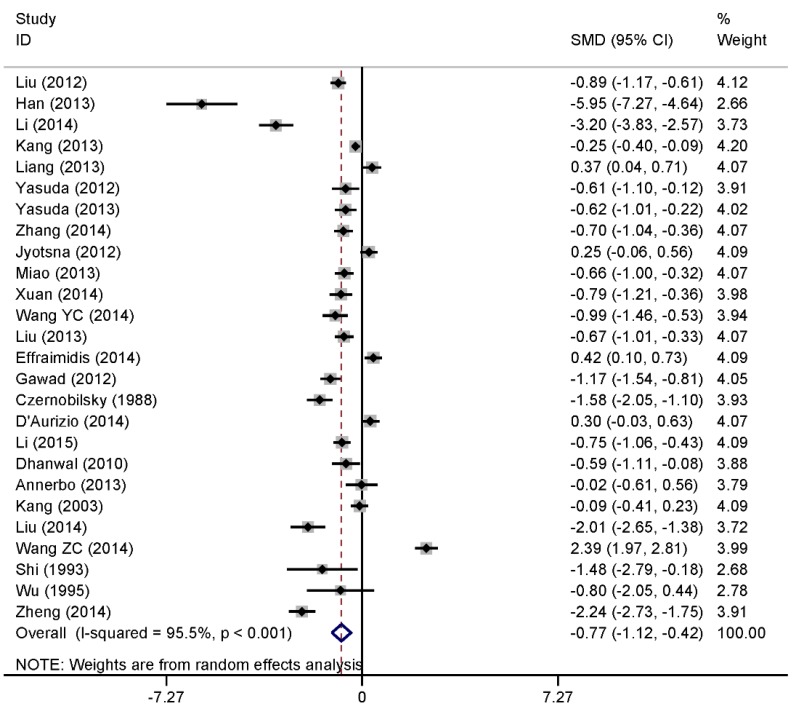
Forest plot of the studies comparing the association between vitamin D levels and Graves’ disease by meta-analysis with the random effects analysis. SMD, standardized mean difference.

The analysis included 26 studies involving 1748 cases and 1848 controls. Combining effect sizes from 26 studies for Graves’ disease as an outcome found a pooled effect of SMD = −0.77 (95% CI: −1.12, −0.42; *p* < 0.001) favoring the low vitamin D level of patients with Graves’ disease by the random effect analysis (Figure 2), which was statistically significant compared to the control group. However, a high degree of heterogeneity was found (*I*^2^ = 95.5%, *p* < 0.001).

### 3.3. Subgroup Analysis

**Figure 3:**
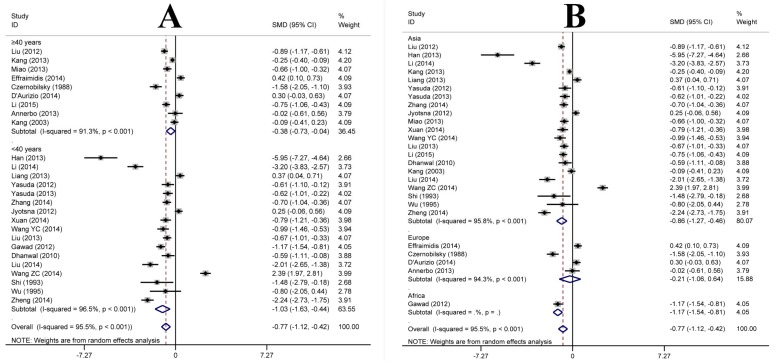

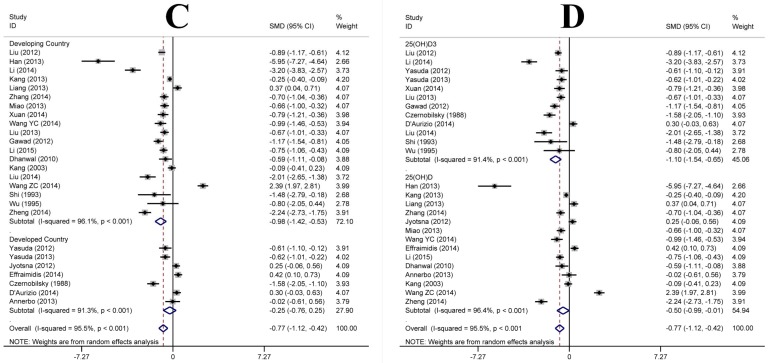

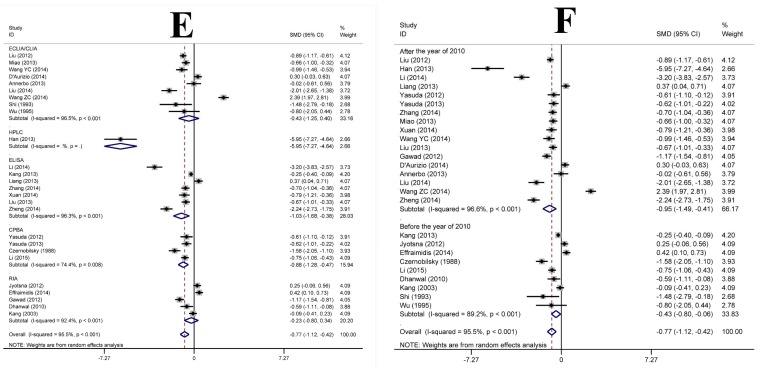
Forest plot of the studies comparing the association between vitamin D levels and Graves’ disease in the subgroups by meta-analysis with the random effects analysis. SMD, standardized mean differences; (**A**) mean age group; (**B**) geographic location; (**C**) country type; (**D**) detection index; (**E**) assay method; (**F**) study period.

We performed subgroup analysis to analyze the sources of heterogeneity (Figure 3). Six factors were used for subgroup analysis, including mean age of case group (≥40/<40 years) (Figure 3A), geographic location (Asia/Europe/Africa) (Figure 3B), country type (developed/developing) (Figure 3C), detection index (25(OH)D/25(OH)D_3_) (Figure 3D), assay method (ELISA/ECLIA/RIA/CPBA/HPLC) (Figure 3E) and study period (before/after 2010) (Figure 3F). Figure 3 A–F shows that the heterogeneity remained high (>80%) in all subgroups, although a slight decrease occurred in the subgroups of assay method (Figure 3E). Interestingly, the SMDs were −0.21 (95% CI: −1.06, 0.64) in Europe, −0.86 (95% CI: −1.27, −0.46) in Asia, and −1.17 (95% CI: −1.54, −0.81), respectively.

**Table 1:** The characteristics of the included studies in the meta-analysis.

No.	First Author	Year	Country	Study Year	Age of Patients (Mean ± SD)	Detection Index	Assay Method	Sample Size (Case/Control)	Quality Score *
1	Li *et al.* [26]	2015	China/Jiangsu	2010	41 ± 10	25(OH)D	CPBA	128/60	9
2	Li *et al.* [5]	2014	China/Shanxi	2011–2012	34 ± 14	25(OH)D_3_	ELISA	40/50	8
3	Zhang *et al.* [13]	2015	China/Hunan	2012–2012	34 ± 12	25(OH)D	ELISA	70/70	8
4	Xuan *et al.* [10]	2014	China/Jiangsu	2013–2014	33 ± 12	25(OH)D_3_	ELISA	47/45	7
5	Wang Y.C. *et al.* [8]	2014	China/Anhui	2013	35 ± 8	25(OH)D	ECLIA	60/30	7
6	Effraimidis *et al.* [24]	2012	Netherland	2003	42 ± 13	25(OH)D	RIA	78/78	9
7	D’Aurizio *et al.* [22]	2015	Italy	2014	47 ± 16	25(OH)D_3_	CLIA	48/126	9
8	Liu *et al.* [27]	2014	China/Hebei	2013	34 ± 11	25(OH)D_3_	ECLIA	35/24	9
9	Wang Z.S. *et al.* [9]	2014	China/Hainan	2012–2013	32 ± 5	25(OH)D	ECLIA	62/91	7
10	Zheng *et al.* [17]	2014	China/Zhejiang	2010–2011	36 ± 8	25(OH)D	ELISA	72/39	7
11	Han *et al.* [16]	2013	China/Guangdong	2012–2013	36 ± 7	25(OH)D	HPLC	30/20	7
12	Kang *et al.* [14]	2013	China/Shandong	2009–2010	43 ± 8	25(OH)D	ELISA	280/439	7
13	Liang *et al.* [31]	2013	China/Hunan	2012–2012	34 ± 12	25(OH)D	ELISA	70/70	9
14	Yasuda *et al.* [12]	2013	Japan	2011	38 ± 7	25(OH)D_3_	CPBA	54/49	8
15	Miao *et al.* [7]	2013	China/Liaoning	2011–2012	40 ± 15	25(OH)D	ECLIA	70/70	9
16	Liu *et al.* [28]	2013	China/Jiangsu	2011–2012	37 ± 11	25(OH)D_3_	ELISA	118/50	9
17	Annerbo *et al.* [20]	2014	Sweden	2009–2012	41 ± 14	25(OH)D	ECLIA	56/14	9
18	Liu *et al.* [6]	2012	China/Henan	2010–2011	42 ± 9	25(OH)D_3_	ECLIA	80/165	8
19	Yasuda *et al.* [11]	2012	Japan	2011	37 ± 13	25(OH)D_3_	CPBA	26/46	8
20	Jyotsna *et al.* [4]	2012	India	2006–2008	36 ± 11	25(OH)D	RIA	80/80	7
21	Abd El Gawad *et al.* [19]	2012	Egypt	2011	38 ± 5	25(OH)D_3_	RIA	90/55	9
22	Kivity *et al.* [15]	2011	Israel	2006	45 ± 16	25(OH)D	ECLIA	22/98	8
23	Dhanwal *et al.* [23]	2010	India	2010	34 ± 9	25(OH)D	RIA	30/31	8
24	Kang *et al.* [25]	2003	China/Tianjin	2000	45 ± 12	25(OH)D	RIA	74/80	7
25	Wu *et al.* [30]	1995	China/Shanghai	1990	NA	25(OH)D_3_	ECLIA	6/5	6
26	Shi *et al.* [29]	1993	China/Shanghai	1991	32 ± 4	25(OH)D_3_	ECLIA	6/6	7
27	Czernobilsky *et al.* [21]	1988	Germany	1988	40 ± 10	25(OH)D_3_	CPBA	38/55	9

ELISA, enzyme-linked immunosorbent assay; HPLC, high performance liquid chromatography; ECLIA, electrochemiluminescence immunoassay; CLIA, chemiluminescent immunoassay method; CPBA, competitive protein binding assay; RIA, radioimmunoassay; NA, no data in the reference; SD, standard deviation; * The quality score was evaluated by the Cochrane’s Newcastle-Ottawa Scale evaluation standard for case-control study.

### 3.4. Meta Regression Analysis

A meta-regression was performed further to explore the possible sources of the heterogeneity. We put the six factors in the subgroup analysis into the meta-regression. As shown in Table 2, none of the six factors had any definite influence on heterogeneity except for assay method (*p* = 0.048).

**Table 2:** Meta-regression of the six factors.

Factor	Coefficient	SE	95% CI		*t*	*p*
Age	−0.18	0.65	−1.54	1.17	−0.29	0.779
Geographic location	0.11	0.6	−1.15	1.37	0.18	0.858
Country type	−1.33	0.73	−2.85	0.19	−1.83	0.083
Detection index	−0.60	0.58	−1.80	0.61	−1.04	0.312
Assay method	−0.58	0.27	−1.16	−0.01	−2.11	0.048
Study period	−0.53	0.64	−1.86	0.81	−0.82	0.420
Constant	4.75	2.33	−0.13	9.63	2.04	0.056

SE, standard error; CI, confidence interval; *t*, *t*-value; *p*, *p*-value.

### 3.5. Sensitivity Analysis

**Figure 4:**
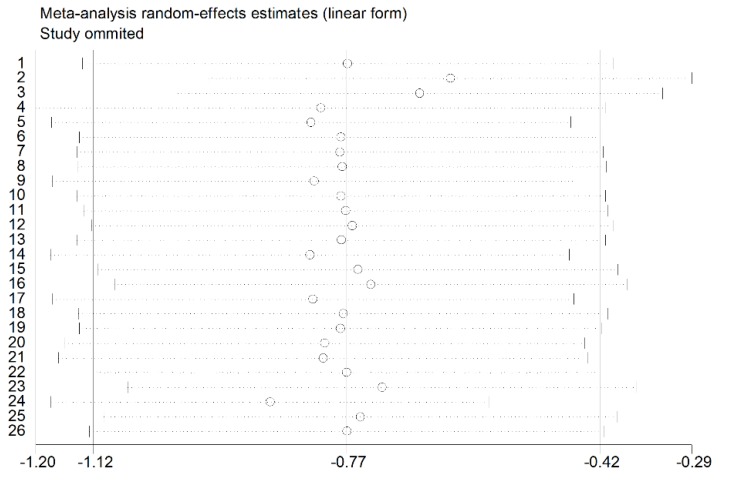
The sensitivity analysis for the association between vitamin D levels and Graves’ disease by the random effects analysis.

The sensitivity analysis was performed and shown in Figure 4, which demonstrated stability and reliability of the meta-analysis results through consistency of meta-analysis results and between the different subgroups. Observed from the Figure 4, the omitted studies by Han *et al.* and Wang *et al.* resulted in the greater change of the estimated values compared to other studies, respectively. However, the significant relationships between the low level of vitamin D and Graves’ disease in all of the situations were evaluated.

### 3.6. Publication Bias

**Figure 5:**
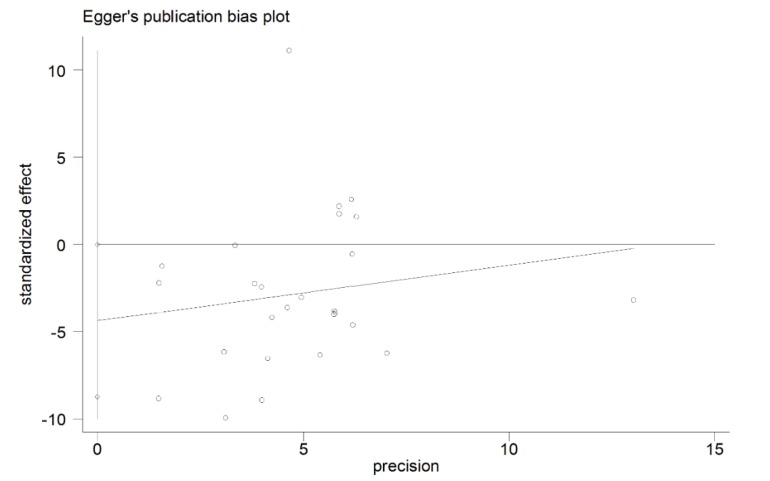
The Egger’s publication bias plot for the association between vitamin D levels and Graves’ disease.

Figure 5 showed the Egger’s publication bias plot in the meta-analysis. The plots shape, as well as the *p* value from Egger’s regression (*p* = 0.049), did not show strong evidence of publication bias.

### 3.7. Categorical Data of Vitamin D Level and Graves’ Disease

**Figure 6:**
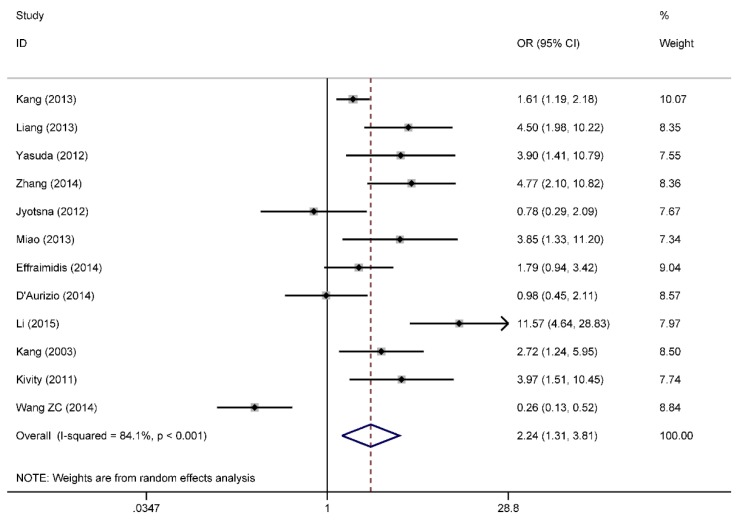
Forest plot of the studies comparing the association between vitamin D deficiency and Graves’ disease by meta-analysis with the random effects analysis. OR, odds ratio.

**Figure 7:**
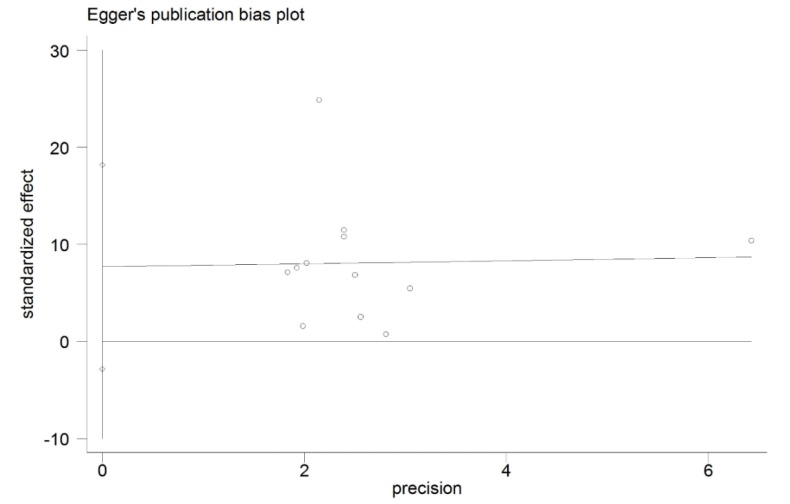
The Egger’s publication bias plot for the association between vitamin D deficiency and Graves’ disease.

In total, 13 studies were included to analyze the association between vitamin D deficiency and Graves’ disease. Due to the result of sensitivity analysis, the study by Han *et al.* was excluded. The patients with Graves’ disease were more likely to be deficient in vitamin D compared to the controls (OR = 2.24, 95% CI: 1.31, 3.81) with a high heterogeneity (*I*^2^ = 84.1%, *p* < 0.001) in Figure 6. We did not find the publication bias from the Egger’s regression (*p* = 0.136) in Figure 7.

## 4. Discussion

We further analysed the relationship between vitamin D levels and Graves’ disease when including more references on the basis of the previous meta-analysis by Wang *et al.* [18]. We also explored the sources of high heterogeneity, finding that the assay method partly contributed to it.

Most current evidence confirmed a higher prevalence of low vitamin D levels or deficiency in patients with Graves’ disease [5,6,10,11,12,14]. In our meta-analysis, which suggested the actual refinement relationship, the absolute SMD value and the OR value were lower than those in the study by Wang *et al.* [18], respectively. We also found that a larger significant difference between the vitamin D levels and Graves’ disease was found in Africa compared to Asia, while no significant difference was found in Europe, which may be due to the living standard and economic level. However, as Rotondi *et al.* noted, the existence of an association, even when supported by strong statistical significance, does not automatically imply that a causal relationship exists [33]; it is therefore necessary to perform more cohort or experimental studies to confirm the causality.

The meta-regression analysis showed the assay method contributed to the heterogeneity. However, the heterogeneity revealed only a slight drop in the subgroup analysis of the assay method. The high heterogeneity may have also arisen due to other reasons, which could not be analyzed in the study because of the partial loss of data or unrecognizable details, such as the characteristics of study subjects, differences in the operating protocol, and so on.

Han *et al.* reported that the vitamin D level in the case group was obviously lower than that in the control group (58.84 ± 8.01 ng/mL) [16], which was much higher than in other studies. This may be related to the sample from healthy control or the assay method (HPLC) in the study. In the meta-analysis on the categorical data, we omitted this study to avoid the significant influence on the result when analyzing the categorical data.

Certain limitations were present in our study. We did not obtain the data of vitamin D deficiency in the references with the vitamin D levels from the authors. The strength of our study was the fact that we used a relatively wide range of references to determine the association between vitamin D and Graves’ disease and reduced the publication bias, as well as improved the accuracy of estimating the risk.

In summary, we further confirmed that low vitamin D status may increase the risk of Graves’ disease. However, whether vitamin D deficiency favor the onset of the disease or supplement of vitamin D has any beneficial therapeutic effect in Graves’ disease needs to be resolved.
